# A simulation study of the system characteristics for a long axial FOV PET design based on monolithic BGO flat panels compared with a pixelated LSO cylindrical design

**DOI:** 10.1186/s40658-023-00593-0

**Published:** 2023-12-01

**Authors:** Meysam Dadgar, Jens Maebe, Maya Abi Akl, Boris Vervenne, Stefaan Vandenberghe

**Affiliations:** 1 Department of Electronics and Information Systems, Medical Image and Signal Processing, Ghent University, C. Heymanslaan 10, Ghent, Belgium; 2 Division of Arts and Sciences, Texas A&M University at Qatar, Doha, Qatar

**Keywords:** Total-body, Monolithic, Walk through PET, Biograph vision quadra

## Abstract

****Background**:**

Although a new generation of tomographs with a longer axial field-of-view called total-body PET have been developed, they are not widely utilized due to their high cost compared to conventional scanners. The newly designed walk-through total-body PET scanner is introduced as a high-throughput and cost-efficient alternative to total-body PET scanners, by making use of a flat panel geometry and lower cost, depth-of-interaction capable, monolithic BGO detectors. The main aim of the presented study is to evaluate through Monte Carlo simulation the system characteristics of the walk-through total-body PET scanner by comparing it with a Quadra-like total-body PET of similar attributes to the Siemens Biograph Vision Quadra.

****Methods**:**

The walk-through total-body PET is comprised of two flat detector panels, spaced 50 cm apart. Each panel, 70 $\times$ 106 cm$^2$ in size, consists of 280 BGO-based monolithic detectors. The Quadra-like TB-PET has been simulated based on the characteristics of the Biograph Vision Quadra, one of the most common total-body PET scanners with 106 cm of axial field-of-view, which is constructed with pixelated LSO scintillation crystals. The spatial resolution, sensitivity, count rate performance, scatter fractions, and image quality of both scanners are simulated in the GATE simulation toolkit for comparison.

****Results**:**

Due to the DOI-capable detectors used in the walk-through total-body PET, the values of the spatial resolution of this scanner were all below 2 mm along directions parallel to the panels, and reached a maximum of 3.36 mm in the direction perpendicular to the panels. This resolution is a large improvement compared to the values of the Quadra-like TB-PET. The walk-through total-body PET uses its maximum sensitivity (154 cps/kBq) for data acquisition and image reconstruction.

****Conclusion**:**

Based on the combination of very good spatial resolution and high sensitivity of the walk-through total-body PET, along with a 2.2 times lower scintillation crystal volume and 1.8 times lower SiPM surface, this scanner can be a very cost-efficient alternative for total-body PET scanners in cases where concomitant CT is not required.

## Background

Positron emission tomography (PET) is an imaging modality widely used in oncology, cardiology, and neurology to investigate noninvasively in vivo a variety of cellular and molecular processes [1]. Due to the value of PET in diagnosing oncological abnormalities, it has become a common imaging modality in this field.

Despite the advantages and wide applications of PET scanners as a diagnostic imaging modality, conventional models have barriers that limit their applicability [2]. In a conventional PET/CT scanner, a so-called short axial field-of-view (AFOV) PET, only 15–35 cm of the patient’s body can be imaged at once [1]. However, since we typically require an image of the torso or full body (e.g., for melanoma cases) to evaluate the presence of metastases, the acquisition process needs to be repeated at multiple bed positions, which increases scan times. This requires the patient to be translated on a table (bed) through the scanner bore. In addition, the limited axial length of standard PET/CT scanners results in limited sensitivity since only a very small fraction (<1$\%$) of the isotropically emitted photon pairs both fall within this short axial FOV and are stopped by the detectors [1, 3]. Poor scanner sensitivity results in images with a lower signal-to-noise ratio, which can be improved by longer scan times or higher injection doses. This is, however, usually not possible.

Recent evolution in PET/CT imaging explores the potential of having a longer AFOV or a so-called total-body (TB) PET scanner to improve sensitivity [3–5]. The idea is to have detector coverage over the whole torso (about 1 m axial length, e.g., the Biograph Vision Quadra [6, 7]) or even the “total-body” (about 2 m axial length, e.g., the uExplorer [3, 8]). This allows for a wider acceptance angle and therefore increased sensitivity and simultaneous imaging of multiple organs.

While long AFOV systems open up very promising clinical applications [9], there are some major challenges that limit their implementation into more routine clinical use in a standard nuclear medicine department. Current TB-PET scanners come at high acquisition and installation costs [3]: extending imaging systems in the axial direction implies that more detectors are used to cover the patient over the longer axial length (e.g., 4x more detectors for 1m AFOV). After certain fixed costs such as the CT component, bed, and installation are taken into account, the bulk of the scanner price scales linearly with the number of detectors used.

This issue inspired extensive efforts to develop affordable alternative systems by research groups around the world. Possible approaches include sparse configurations to reduce the total number of detectors while maintaining a long AFOV [10], the use of alternative, cheaper scintillation materials such as BGO [9] or plastic scintillators [11], and monolithic detector blocks [12, 13].

We propose a novel design concept for affordable TB-PET to go toward faster and lower dose imaging with higher (more efficient) patient throughput at a lower component cost: a flat panel high-resolution walk-through (WT) TB-PET design with 106 cm AFOV [14–17]. As patient positioning on/off the scanner bed becomes the dominant factor in limiting practical throughput in Quadra-like TB-PET, the proposed design consists of two flat detector panels with patients standing upright in between the panels. The setup process for a scan is very quick as patients no longer need to be positioned on the bed and instead walk into the scanner. The panels can translate up and down to adapt to patient height, and offer the capability of whole-body scanning if required. The design uses monolithic BGO scintillation crystals, reducing component cost compared to pixelated L(Y)SO detectors [18]. In addition, BGO has a higher stopping power (i.e., sensitivity), and monoliths offer intrinsic depth-of-interaction (DOI) decoding capabilities together with high (1–1.5 mm range) detector spatial resolution. The primary drawback of BGO is its lower time-of-flight (TOF) resolution, although by integrating deep learning into the detector readout, the coincidence time resolution (CTR) can be improved considerably [18].

The main aim of the presented research is to investigate through simulation the system characteristics of the WT-PET and compare it with a Quadra-like total-body PET of similar attributes to the Siemens Biograph Vision Quadra, as both scanners have the same AFOV (106 cm).

## Methods

### System specifications

**Fig. 1:**
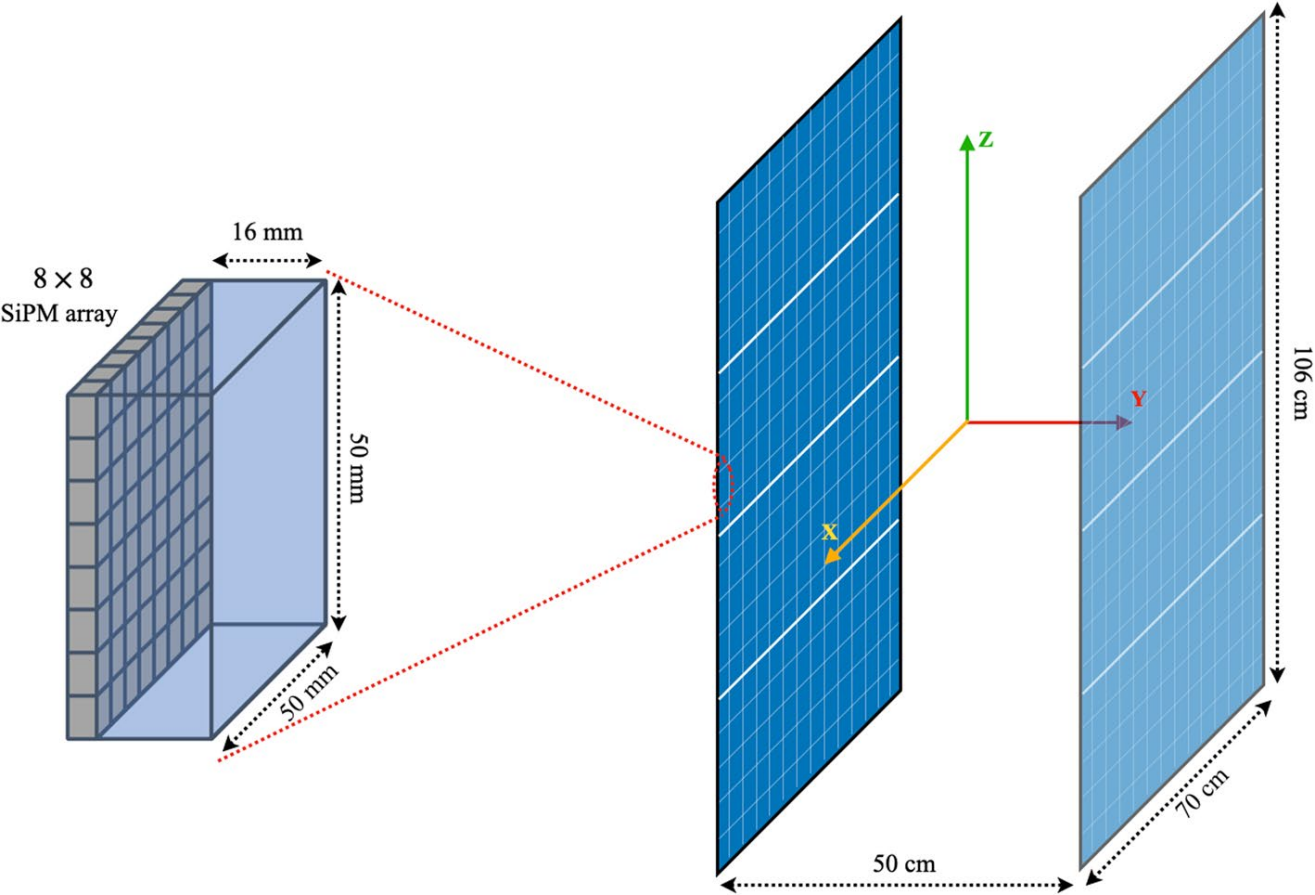
Schematic illustration of the (left) single BGO monolithic detector coupled with a 8 $\times$ 8 SiPM array. (Right) The detector comprised two flat panels, each one consists of 14 $\times$ 20 monolithic detectors with the possibility of performing the scan in the stand position

The WT-PET design consists of two vertically positioned flat detector panels spaced 50 cm apart, each 70 cm wide and 106 cm high, as shown in Fig. 1. The AFOV (106 cm) is sufficient for simultaneous head and torso imaging, and total-body imaging can be achieved by sliding the panels down during an acquisition.

Given the flat panel design, the detectors can be positioned much closer to the patient, reducing the number of detectors. Moreover, the WT-PET scanner will use newly developed monolithic BGO detectors [18]. The scintillator blocks are 50$\times$50$\times$16mm$^3$ in size and coupled to an 8$\times$8 array of SiPMs. A total of 14 $\times$ 20 = 280 detectors are used per panel. The detectors provide a 2D spatial resolution of 1.3 mm full width at half maximum (FWHM), a DOI resolution of 2 mm FWHM and a coincidence time resolution (CTR) of 327 ps FWHM [18]. These are average (measured) values, with the effects of crystal scatter already included. The combination of fewer detectors and overall cost-efficient detector technology brings the price of the proposed WT-PET design close to the price of a standard PET/CT scanner.

The main system specifications and simulation parameters used for the WT-PET system are shown in Table 1, next to the Quadra-like TB-PET for comparison.

**Table 1:** System characteristics of the WT-PET and the Quadra-like TB-PET

System parameters	WT-PET	Quadra-like TB-PET
Scintillator material	BGO	LSO
Crystal size (mm)	$50\times 50\times 16$	$3.2\times 3.2\times 20$
DOI capable	Yes	No
Ring diameter (cm)	–	82
Panels distance (cm)	50	–
Axial FOV (cm)	106	106
Shortest LOR through the center (cm)	50	82
Longest LOR through the center (cm)	138.61	134.00
Energy resolution	15 %	11 %
Energy window (keV)	434-645	455-645
TOF resolution (ps)	327	228
Dead time (ns)	370	320

### Monte Carlo simulation and image reconstruction

Simulation of PET acquisitions is done using the Geant4 Application for Tomographic Emission (GATE) Monte Carlo software [19, 20]. GATE includes coincidence sorting, producing the listmode (LM) data that is later used for image reconstruction. All simulations, except for count rate performance and spatial resolution measurements, are performed for 30 seconds of data acquisition, since that is the high-throughput scan time that is being aimed for in the WT-PET design. In the case of the Quadra-like TB-PET system, measurements are done both without a maximum ring difference cut (322 MRD), as well as with a cut of 85 rings applied (85 MRD) [21]. A coincidence time window (CTW) of 5 ns is chosen for the WT-PET, whereas the Quadra-like TB-PET uses a CTW of 4.7 ns. The digitizer in the GATE simulation of the investigated scanners used the alternative coincidence sorter (enabled with allDigiOpenCoincGate = true) and the “takeAllGoods” multiples policy. Note that the simulation of the Quadra-like TB-PET is based on the physical geometry of the scanner, but certain differences could remain between the simulation and experiment in terms of coincidence processing and image reconstruction.

For image reconstruction, a locally developed image reconstruction software written in C++, called Quantitative Emission Tomography Iterative Reconstruction (QETIR), is used. QETIR includes maximum-likelihood expectation maximization (MLEM) and ordered subset expectation maximization (OSEM) algorithms for iterative listmode reconstruction. QETIR, as a flexible image reconstruction software, can be used with various scanner configurations [11, 22, 23]. Depending on the purpose of the reconstruction, both TOF LM and non-TOF LM can be utilized. In this study, all reconstructions are done with TOF, using no subsets. No regularization is included, nor is any post-processing done on the final reconstruction.

**Fig. 2:**
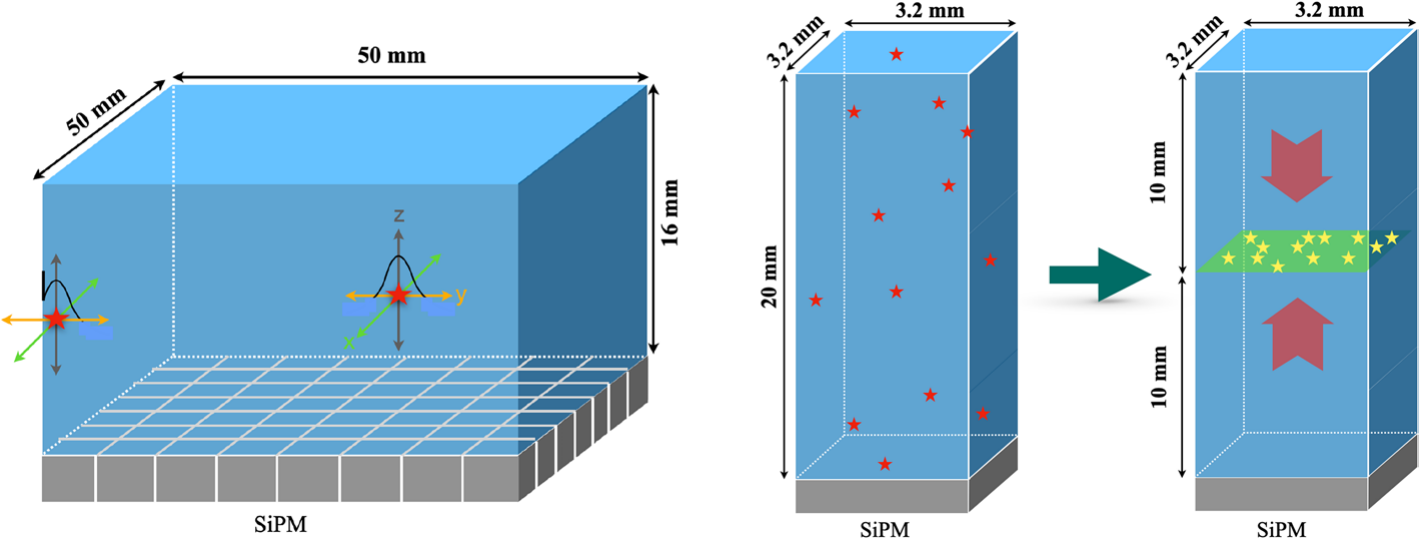
(Left) A single monolithic detector utilized in the WT-PET system where the ground-truth interaction point of the gamma photon (red star) is smeared along the *X*, *Y*, and *Z* axes. (Right) Schematic illustration of the smearing applied to interaction points of gamma photons (red stars) within a single scintillation crystal pixel as the smallest detection unit in the Quadra-like TB-PET

We know the exact interaction point of the gamma photons with the scintillator from the GATE simulation. Note that in case of crystal scatter, GATE returns an energy-weighted interaction point, which is a good estimate for monolithic detectors since positioning is done based on the light spread. To obtain realistic results however, it is essential to smear this interaction point. For the case of the WT-PET, since it is equipped with DOI-capable monolithic detectors, the interaction point of the gamma photon within the BGO scintillation crystal is smeared with a Gaussian in 3D, as shown in Fig. 2. This is a fair approximation for the spatial resolution in a monolithic detector, except for interactions close to the edges or close to the SiPM array, where due to surface reflections or lack of light spread a certain bias may be introduced, asymmetrically degrading the resolution [12]. The values used for smearing are equal to the average detector spatial resolution in each direction, that is $\sigma _{x,y} =0.55$ mm (2D spatial resolution of $\sim$ 1.3 mm FWHM) and $\sigma _{z} = 0.85$ mm (DOI resolution of $\sim$ 2 mm). These are values obtained for the whole detector, so that the aforementioned discrepancies with the physical detector should average out. If the smearing would place the interaction point outside of the scintillator, the interaction point is instead placed on the very edge of the detector to disallow non-physical LORs. This will introduce some bias (which is not necessarily representative of the physical detector), but the frequency of such an occurrence is rather small given the detector volume relative to its spatial resolution.

As the Quadra-like TB-PET makes use of non-DOI-capable pixelated detectors, a different method of uncertainty in the interaction point is utilized. In this case, all the interaction points within a detector pixel are shifted to the central plane along the depth of the pixel, and are then uniformly randomized within that plane, as shown in Fig. 2.

### Performance measurements

#### Spatial resolution

To estimate the spatial resolution of the WT-PET and the Quadra-like TB-PET systems, six F-18 point sources with a diameter of 0.5 mm and an activity concentration of 15.28 MBq/ml are simulated according to the NEMA NU2-2018 guidelines [24–26]. Three points are located at the central transverse slice of the scanners (1, 10, and 20 cm in the radial direction), and another three at 3/8 of the AFOV. The aforementioned point sources were simulated in a warm background to account for the utilized iterative reconstruction algorithm. Due to the cylindrical geometry of conventional PET scanners, these coordinates provide an estimation of the spatial resolution for any angle around the scanner axis. Given the unique configuration of the WT-PET however, an additional series of six F-18 point sources with similar radial and axial arrangements are simulated, rotated 90 degrees around the scanner axis.

**Fig. 3:**
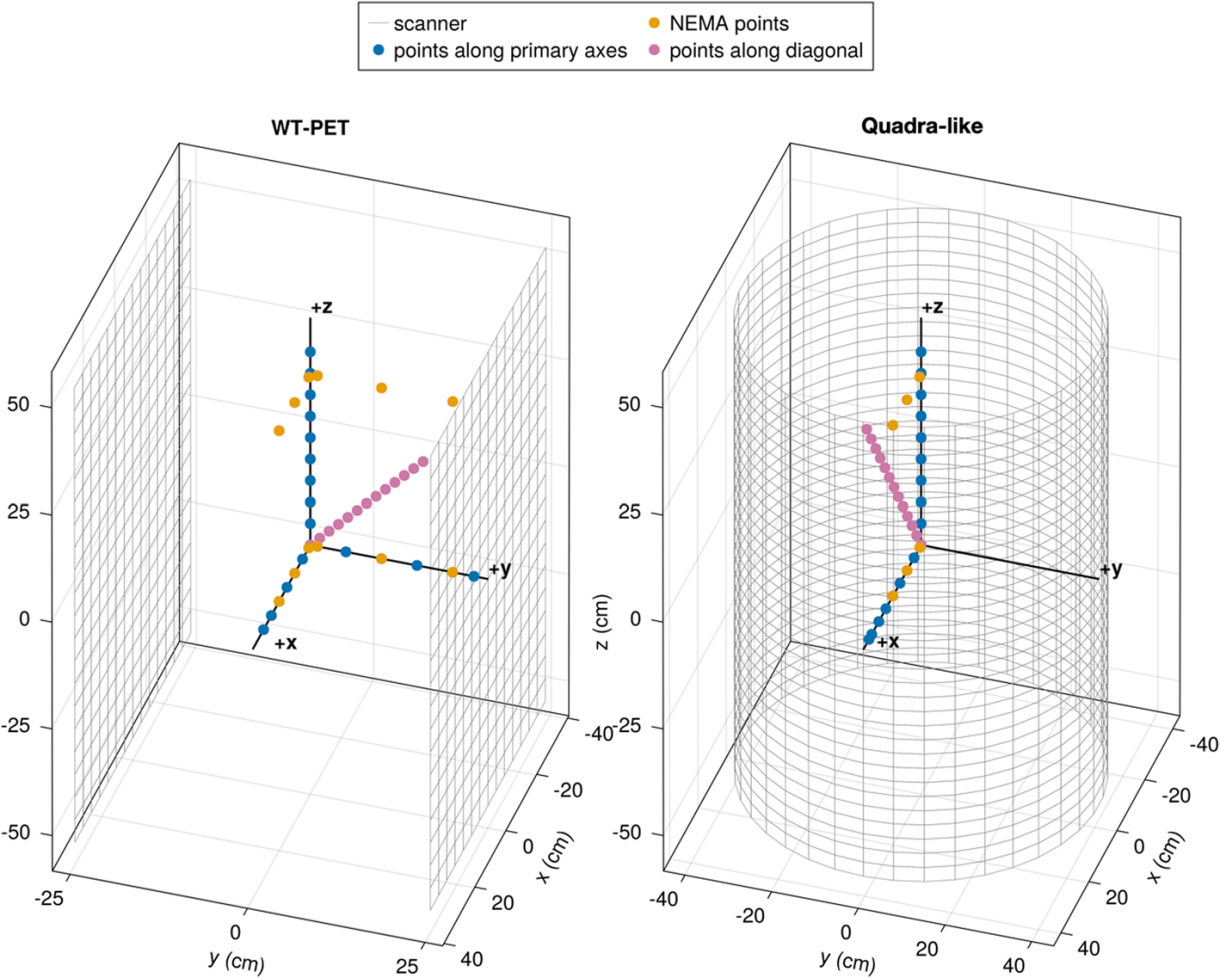
Schematic Illustration of point source locations for the WT-PET (left) and the Quadra-like TB-PET (right). For the WT-PET, the diagonal point sources (pink) are positioned in 3D toward the corner of one panel, while for the Quadra-like TB-PET, the symmetrical configuration results in diagonal point sources confined to the *XZ* plane

To better map the spatial resolution of the scanners under investigation, additional point sources outside of the NEMA guidelines are simulated along specific axes of the scanners, as shown in Fig. 3. In the case of the Quadra-like TB-PET, point sources are placed on the radial, axial, and diagonal (longest possible LOR) axes in steps of 5 cm. For the WT-PET, they are placed on the *X*, *Y*, *Z*, and diagonal axes, again in 5 cm steps. These additional sources were simulated in a cold background for both systems.

Image reconstruction is done with QETIR, based on true coincidences only using the MLEM algorithm (no subsets), 0.5$\times$0.5$\times$0.5 mm$^3$ voxel dimensions and 10 iterations, with no spatial resolution modeling inside the reconstruction.

#### Sensitivity

To evaluate the sensitivity of the WT-PET and the Quadra-like TB-PET, a 70-cm-long F-18 line source with an activity of 1 MBq is placed at the center of each scanner, as well as offset by 10 cm in the radial direction. The line source was surrounded by five concentric aluminum sleeves of the same length. Given the non-cylindrical configuration of the WT-PET system, the line source is offset along two different radial directions, once along the detector panels (X-axis) and once toward the detector panels (Y-axis). Since both the WT-PET and the Quadra-like TB-PET have an AFOV of 106 cm, all simulations are additionally repeated with a 106 cm line source.

#### Count rate performance

The count rate performance of any PET system is essential to evaluate the impact of increasing count rates on image quality. Based on NEMA specifications, count rate simulations for the WT-PET design were performed with a 70-cm-long line source of F-18 in water, at a 4.5 cm offset from the axis of the tomograph, placed in a polyethylene cylinder of 20.3 cm diameter and 70 cm height. Due to the unique configuration of the proposed design, two different offset directions were considered, where the source was placed at $X = 4.5$ cm (shifted laterally, parallel to the panels), and then at $Y = 4.5$ cm (shifted toward one of the panels). For the Quadra-like TB-PET, one source placement at $X = 4.5$ cm was sufficient, given the cylindrical configuration of the detectors. The activity of the line source ranged from 0.045 to 41 kBq/mL, with an acquisition time large enough to ensure that each simulation has a minimum of one million prompt counts. This is followed by analyzing the GATE output files where the true, scatter, and random coincidence counts were sorted based on the GATE tags of event ID and Compton interactions. According to NEMA standards, only data from the central 65 cm of the AFOV was considered, and a mask in the transaxial FOV was applied to set to zero all voxels located further than 12 cm from the center of the scanner. The noise equivalent count rate (NECR) represents an effective true count rate and is defined as:

1 $$\begin{aligned} \textrm{NECR}= \frac{T^2}{T+S+R} \end{aligned}$$

where *T*, *S*, and *R* are the true, scatter and random coincidence count rates, respectively.

#### Scatter fraction

The scatter fraction (SF) measures the relative system sensitivity to scattered radiation. Based on NEMA, the same source and phantom specifications as the NECR study are used to evaluate the SF of both systems. The single slice rebinning (SSRB) algorithm was used for the scatter fraction analysis [27, 28]. The SF is defined as the ratio of scattered events to total events for a low enough count rate at which random rates are below 1% of the true rate [26, 29]. It is expressed as:

2 $$\begin{aligned} \textrm{SF}=\frac{S}{S+T} \end{aligned}$$

#### Image quality

The image quality of the WT-PET and Quadra-like TB-PET is evaluated using the NEMA image quality (IQ) phantom. It comprises of six hot spheres with various diameters (10, 13, 17, 22, 28, and 37 mm) placed in a warm background. The sphere to background activity concentration ratio was 4:1 for both scanners, with a background activity of 5.3 kBq/cc.

To better investigate the impact of limited angle artifacts, additional IQ phantom simulations are performed for the WT-PET, all with two additional lesions (10 mm diameter spheres with 4:1 activity ratio), placed at the edge of the anterior and posterior surfaces of the IQ phantom, mimicking melanoma lesions. In a first study, the IQ phantom positioned at the center of the AFOV is compared to an IQ phantom placed at 1/8 of the AFOV. In a second comparison, we investigate the impact of different TOF values (200, 400, 600, and 800 ps) on the reconstruction. The total acquisition time in all the cases is 30 s.

QETIR is used for image reconstruction. The reconstruction is done based on true coincidences only using the MLEM algorithm (no subsets), 2$\times$2$\times$2 mm$^3$ voxel dimensions and 10 iterations. For both scanners, sensitivity and ground-truth attenuation correction are applied. The quality of the reconstructed image is evaluated based on the contrast recovery coefficient (CRC) calculated using the methodology proposed by NEMA [30].

## Results

### Spatial resolution

**Fig. 4:**
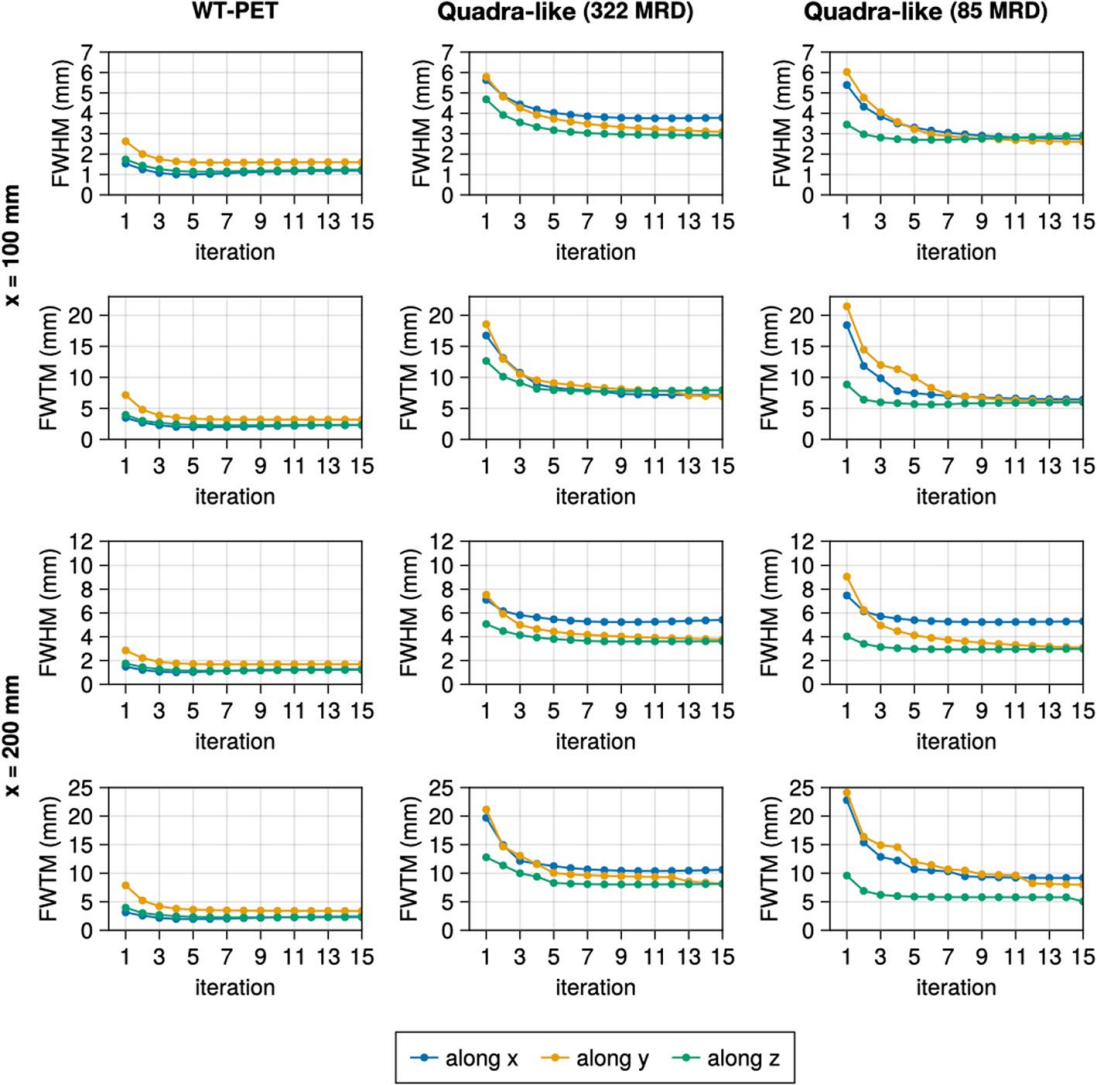
Evolution of FWHM and FWTM for the point sources (in the warm background) at (100, 0, 0) and (200, 0, 0) mm as the function of the number of iterations

Figure 4 shows the spatial resolution of two point sources in a warm back ground, in the central transverse plane, at a radial distance of 10 and 20 cm, in function of the MLEM iteration number. The full width at tenth maximum (FWTM) is provided in addition to the FWHM, given the sometimes non-Gaussian nature of the point source reconstructions [31, 32]. Both FWHM and FWTM were therefore calculated using a linear interpolation between pixels. Based on these figures, we conclude that 10 iterations are sufficient for convergence in both scanners. Therefore, further results are all based on reconstruction with 10 iterations.

**Fig. 5:**
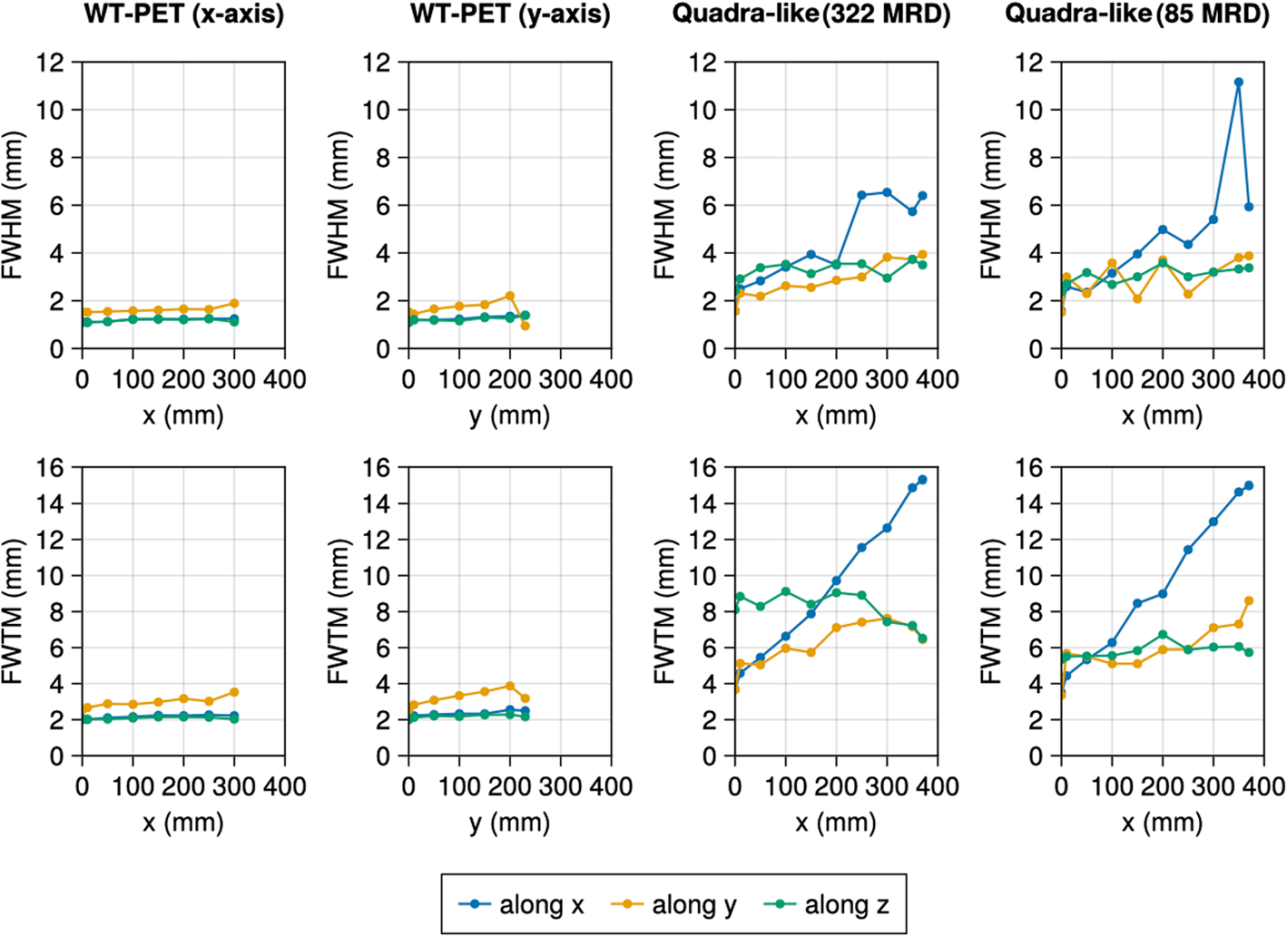
Evolution of the FWHM and FWTM for the non-NEMA point sources (in cold background) located along the radial axis (*X* and *Y* for the WT-PET and *Y* for the Quadra-like TB-PET) at the 10th iteration

**Fig. 6:**
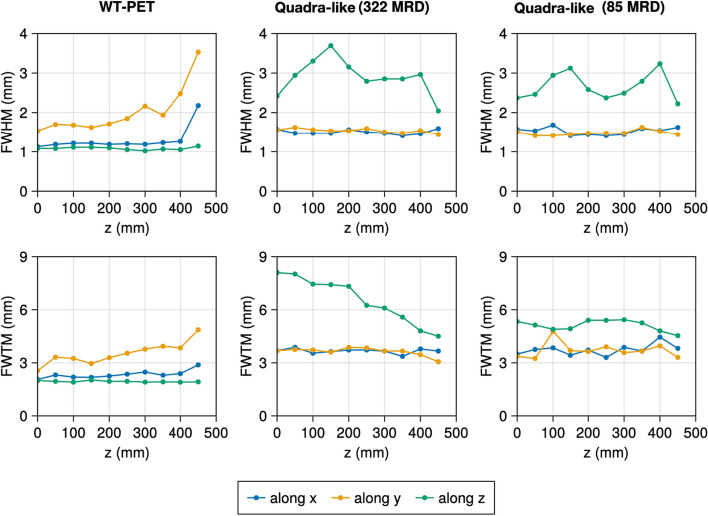
Evolution of the FWHM and FWTM for the non-NEMA point sources (in cold background) located in the axial axis (*Z*) for the WT-PET and Quadra-like TB-PET at the 10th iteration

**Fig. 7:**
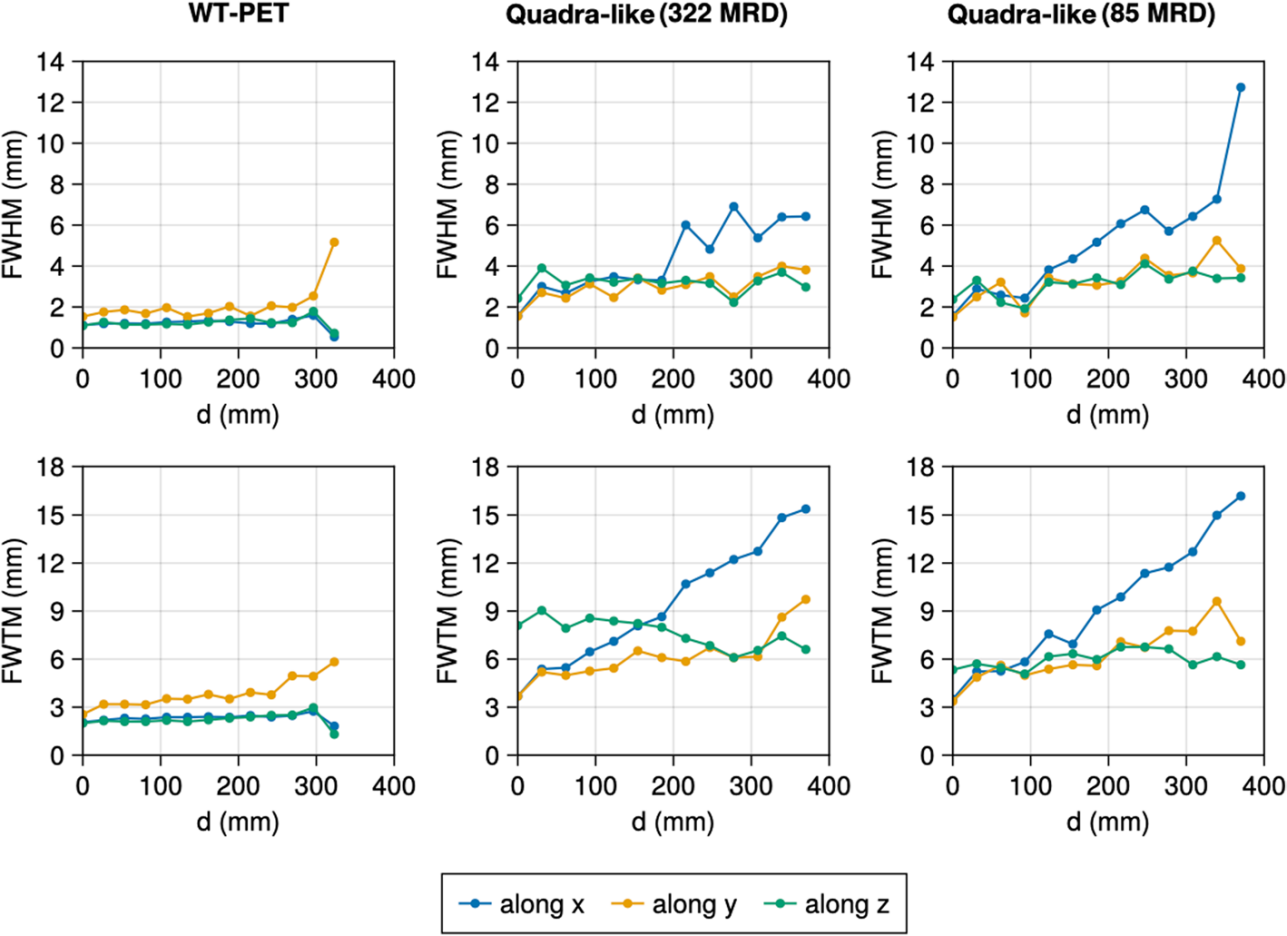
Evolution of the FWHM and FWTM for the non-NEMA point sources (in cold background) located along the diagonal direction (pink points in Fig. 3) for the WT-PET and Quadra-like TB-PET at the 10th iteration

Table 2 shows the spatial resolutions of the NEMA recommended source positions at the 10th iteration with warm background. The spatial resolution of the WT-PET is overall better compared to the Quadra-like TB-PET due to the use of high-resolution DOI-capable monolithic detectors. To investigate the trends in spatial resolution of the WT-PET and Quadra-like TB-PET across their FOV, additional, non-NEMA point sources in a cold background have been simulated. Figure 5 shows the spatial resolution for points located in the radial axis (*X* and/or *Y*), Fig. 6 shows the spatial resolution for points located in the axial axis (*Z*), and Fig. 7 shows the spatial resolution for points located along the diagonal axes of the aforementioned scanners. Since these additional point sources have been simulated in a cold background, their values are not directly comparable to Table 2.

The WT-PET shows a degradation of the spatial resolution for point sources getting closer to the panels ($+Y$), and this degradation is primarily observed for the FWHM/FWTM along *Y*, as shown in Table 2. This is likely due the limited projection angles. For point sources moving to the edge of the scanner, parallel to the panels ($+X$), the spatial resolution remains largely constant. The Quadra-like TB-PET also shows a degradation for point sources getting closer to the detectors ($+X$), but this is now mostly observed for the FWHM/FWTM along both the *X* and *Y* directions (radial and tangential).

**Table 2:** The spatial resolution values of the NEMA point sources in a warm background for the WT-PET and the Quadra-like TB-PET

Scanner	Source position (cm)		FWHM (mm)			FWTM (mm)		
			*x*	*y*	*z*	*x*	*y*	*z*
WT-PET	Center	(1, 0, 0)	1.20	1.62	1.30	2.87	4.14	3.06
		(10, 0, 0)	1.16	1.90	1.12	2.81	4.83	2.89
		(20, 0, 0)	1.17	1.94	1.19	2.60	4.36	2.81
	3/8 of AFOV	(1, 0, 39.75)	1.14	2.13	1.24	2.86	5.22	3.20
		(10, 0, 39.75)	1.21	2.48	1.29	3.19	7.11	3.06
		(20, 0, 39.75)	1.26	2.52	1.05	2.08	5.58	2.08
	Center	(0, 1, 0)	1.18	1.75	1.34	2.43	3.79	2.54
		(0, 10, 0)	1.22	1.92	1.33	2.54	4.20	2.54
		(0, 20, 0)	1.32	2.25	1.41	2.71	7.08	2.71
	3/8 of AFOV	(0, 1, 39.75)	1.29	2.24	1.16	2.58	5.71	2.59
		(0, 10, 39.75)	1.52	2.71	1.46	2.91	6.54	3.35
		(0, 20, 39.75)	1.65	3.36	1.88	3.76	8.18	3.96
Quadra-like TB-PET 322 MRD	Center	(1, 0, 0)	2.55	2.62	2.85	5.68	6.45	7.03
		(10, 0, 0)	3.76	3.27	2.95	7.24	7.98	7.78
		(20, 0, 0)	5.24	3.97	3.62	10.39	9.39	8.03
	3/8 of AFOV	(1, 0, 39.75)	2.09	2.35	2.24	4.76	5.70	4.68
		(10, 0, 39.75)	2.78	3.14	2.89	5.85	7.64	5.03
		(20, 0, 39.75)	4.63	3.31	3.20	8.61	6.81	5.76
Quadra-like TB-PET 85 MRD	Center	(1, 0, 0)	2.62	2.70	2.72	5.02	5.66	4.82
		(10, 0, 0)	2.86	2.72	2.78	6.66	6.43	5.86
		(20, 0, 0)	5.24	3.40	2.94	9.27	9.71	5.79
	3/8 of AFOV	(1, 0, 39.75)	2.22	2.44	2.40	4.80	5.71	4.84
		(10, 0, 39.75	3.31	3.02	2.71	6.35	6.67	5.95
		(20, 0, 39.75)	4.29	2.66	2.23	8.55	10.78	5.82

### Sensitivity

**Fig. 8:**
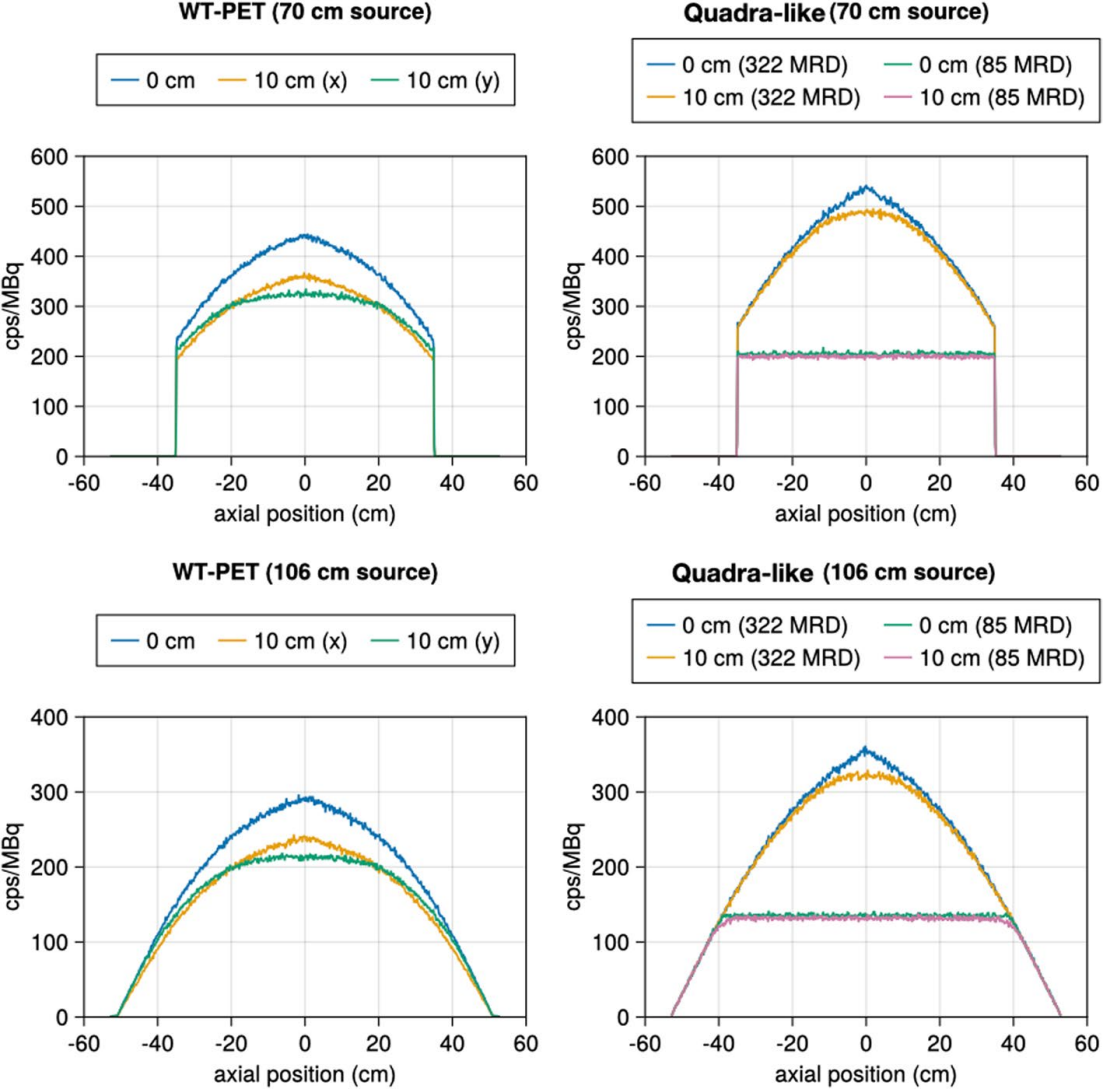
Sensitivity profiles of the WT-PET and Quadra-like TB-PET, both for a 70 cm line source and a 106 cm line source with an activity of 1 MBq

The obtained sensitivity profiles for both scanners are shown in Fig. 8. The Quadra-like TB-PET (applying no MRD cut) reaches a higher sensitivity for both source positions than the WT-PET. This is expected due to the larger detector coverage. The total sensitivities are reported in Table 3.

**Table 3:** The total sensitivity values of the WT-PET and Quadra-like TB-PET for 70 cm and 106 cm length line source

Line source	Sensitivity (cps/kBq)		
	WT-PET	Quadra-like TB-PET	
		85 MRD	322 MRD
70 cm (center)	154.0	87.0	179.7
70 cm (10 cm along *X*)	127.1	84.5	173.2
70 cm (10 cm along *Y*)	124.4	–	–
106 cm (center)	117.2	75.6	137.8
106 cm (10 cm along *X*)	97.0	73.5	133.4
106 cm (10 cm along *Y*)	96.6	–	–

### Count rate performance

**Fig. 9:**
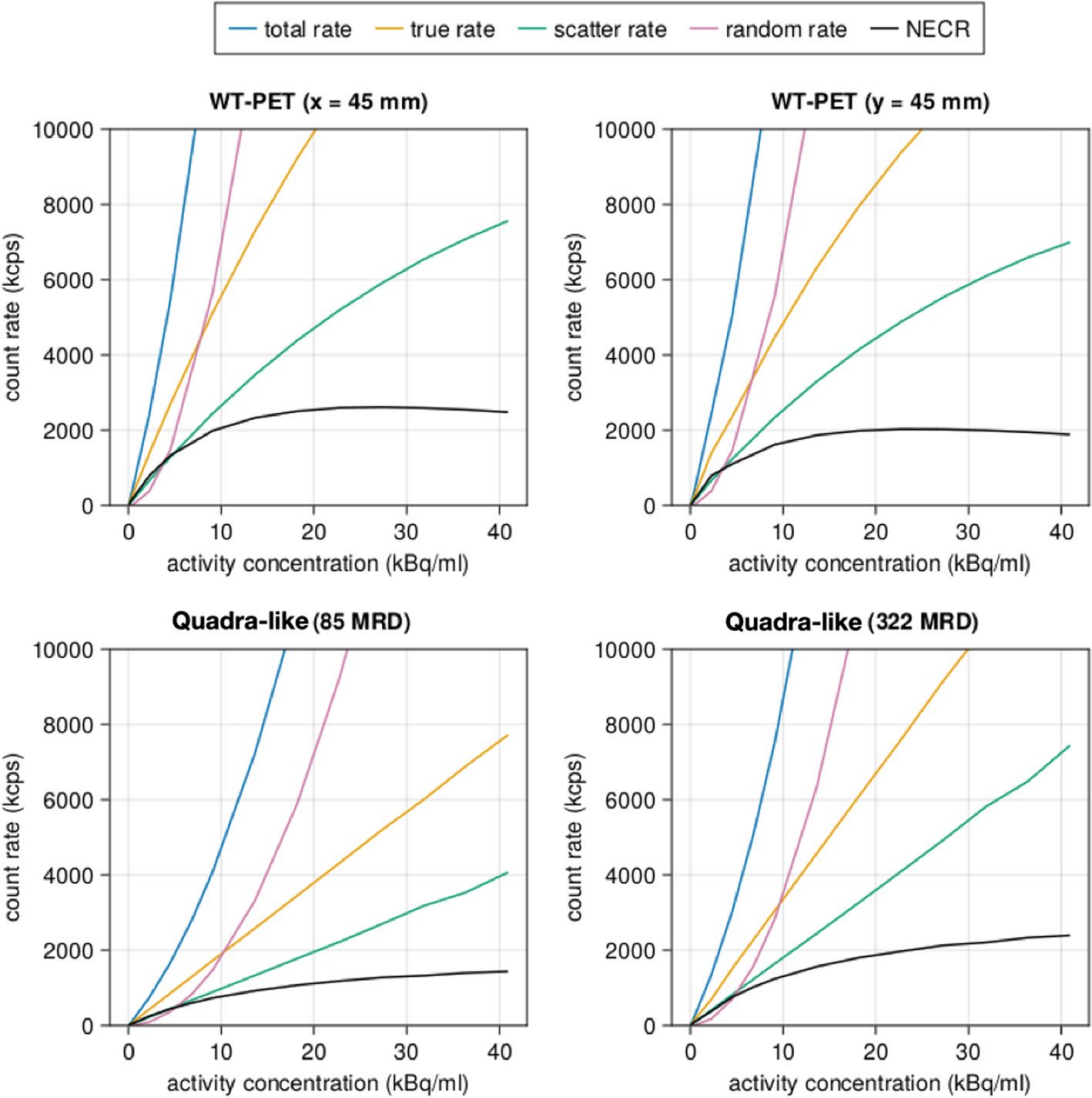
Count rate performance as a function of the activity concentration for the WT-PET and the Quadra-like TB-PET. The source for the WT-PET (CTW = 5 ns) was offset in both the *X* and *Y* directions, and the Quadra-like TB-PET utilized both 322 MRD and 85 MRD (CTW = 4.7 ns)

Figure 9 shows the count rate curves for the WT-PET and the Quadra-like TB-PET. The obtained NECR peaks for the WT-PET, with the line source located at a 45 mm offset from the center in the X and Y direction, are $\sim$ 2.61 kcps and $\sim$ 2.02 kcps at an activity concentration of $\sim$27.3 kBq/ml (x) and $\sim$22.7 kBq/ml, respectively. While for the Quadra-like TB-PET, the NECR peaks are not reached below 40 kBq/ml. Higher activity ranges were not investigated as they are not used in the clinic. The experimental peak value as reported in Prenosil et al. [6] likely arises from a system level bandwidth, which has not been modeled in this study. The WT-PET shows higher count rates compared to the Quadra-like TB-PET in MRD 322, despite its lower sensitivity, which can be explained by the lack of a patient bed.

### Scatter fraction

Table 4 shows the scatter fraction values for the WT-PET and the Quadra-like TB-PET (both 322 and 85 MRD). In the case of the Quadra-like TB-PET, simulations have been done in the presence of the patient bed. Due to the patient’s standing position in the WT-PET, it does not need to be equipped with a bed. This results in an overall lower scatter fraction for the WT-PET.

**Table 4:** Scatter fraction values of the WT-PET and Quadra-like TB-PET

Scanner	Scatter fraction (%)
WT-PET (45 mm offset along *X* axis)	30.72
WT-PET (45 mm offset along *Y* axis)	29.58
Quadra-like TB-PET (with 322 MRD)	36.18
Quadra-like TB-PET (with 85 MRD)	34.80

### Image quality

**Fig. 10:**
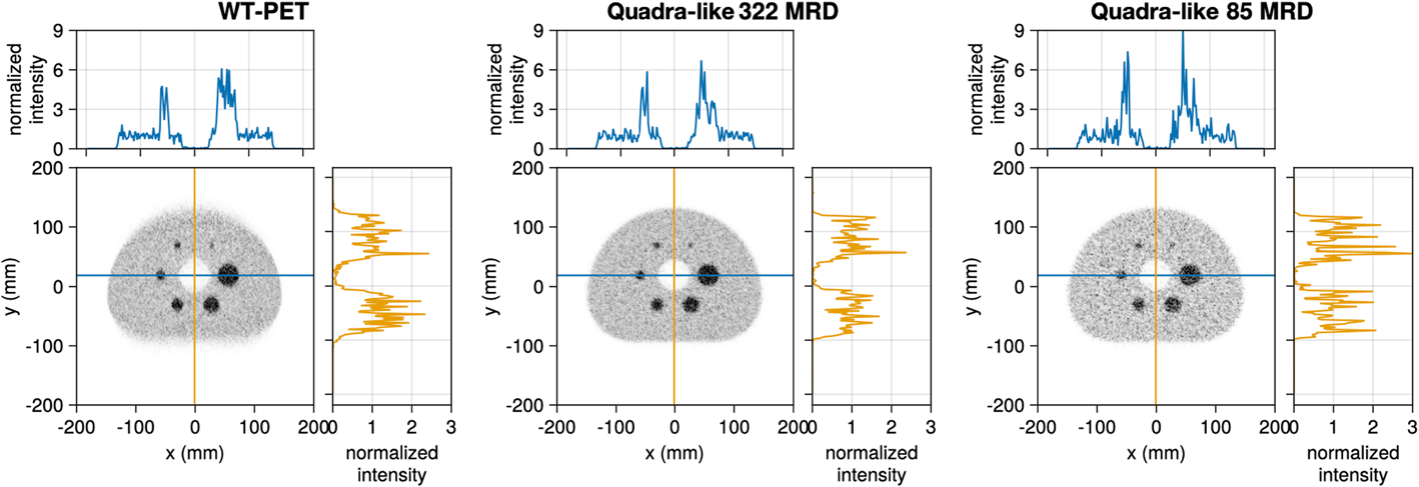
IQ reconstruction at the 10th iteration for the (left) WT-PET, (middle) Quadra-like TB-PET with 322 MRD and (right) Quadra-like TB-PET with 85 MRD. Line profiles are provided with intensity normalized to the average background activity

The reconstructions (10th iteration) of the IQ phantom for both the WT-PET and Quadra-like TB-PET, with 30 s of data acquisition, are shown in Fig. 10. Note that for the WT-PET, the panels are oriented horizontally in this image, above and below the phantom. Some artifacts of the limited projection angles are visible in the reconstruction for the WT-PET, although TOF information constrains these.

**Fig. 11:**
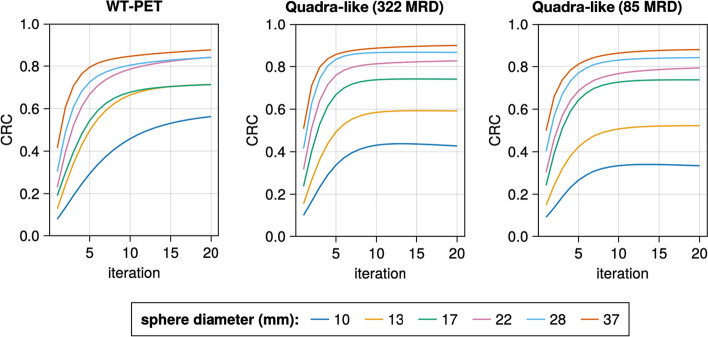
The contrast recovery coefficient (CRC) values of the IQ phantom for both scanners

The image quality is evaluated based on the CRC. Figure 11 shows the CRC values for both scanners, as a function of the iteration number. The largest (37 mm) sphere obtains the highest CRC value in all configurations, and the CRC values improve with higher iteration numbers. However, after roughly the 10th iteration, the CRC value remains largely unchanged, especially for larger spheres and the Quadra-like TB-PET. Note that the values obtained for the Quadra-like TB-PET are lower than the ones presented in [6]. This is primarily due to the considerably lower scan time (30 s) used in our simulation.

**Fig. 12:**
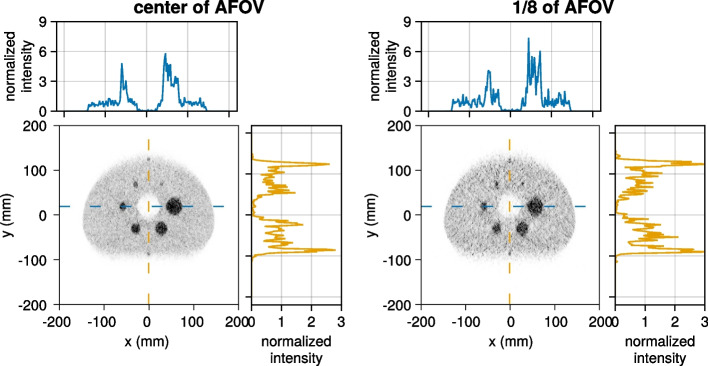
The IQ phantom reconstructed with two extra (10 mm diameter, 4:1 activity ratio) spheres placed at the anterior and posterior surfaces of the IQ phantom. The IQ phantom was placed at the center of the AFOV (left) and at 1/8 of the AFOV (right). The intensity of the line profile is normalized to the average background activity

Figure 12 shows the IQ phantom, with inclusion of additional lesions at the edge of the anterior and posterior surfaces, at the center of the AFOV and 1/8 of the AFOV. The additional lesions are visible in both cases, although as expected the limited angle artifacts (and noise due to reduced sensitivity) are larger for the IQ phantom placed at 1/8 of the AFOV.

**Fig. 13:**
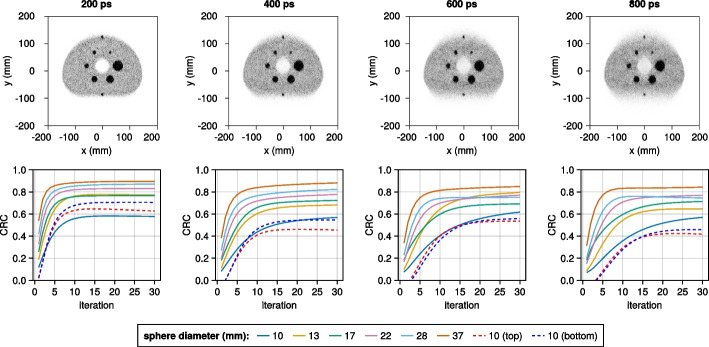
The IQ phantom reconstructed for the WT-PET system with different TOF resolutions (the 10th iteration is shown), and the CRC values for different sphere diameters (4:1 activity ratio) in function of the iteration number

Figure 13 shows the impact of different TOF values, ranging from 200 to 800 ps, on the IQ phantom reconstruction (located at the center of the AFOV for the WT-PET system). Although the limited angle artifacts are more pronounced for higher TOF values, all spheres remain detectable, including the additional lesions.

## Discussion

A comparison of the performance characteristics between the WT-PET and Quadra-like TB-PET system was performed by simulating both scanners’ spatial resolution, NECR, scatter fraction, sensitivity, and image reconstruction quality, adhering to the NEMA NU2-2018 guidelines where possible. Due to the fact that most of the currently available PET scanners have a smaller AFOV (15–35 cm), NEMA NU2-2018 is not fully compatible with larger AFOV tomographs such as TB-PET scanners. For this reason, alongside the standards recommended by NEMA, an extended version of the investigation using an elongated source (106 cm) and additional point source locations was performed.

While the spatial resolution of the Quadra-like TB-PET is more sensitive to the position of the source, the WT-PET offers a more uniform spatial resolution within its entire FOV. The main reason for such a unique performance of the WT-PET is due to the utilization of DOI-capable monolithic detectors in its configuration, as opposed to the non-DOI-capable pixelated detectors used in the Quadra-like TB-PET. It should be noted that the spatial resolution obtained for the simulated Quadra-like scanner is better than what is achieved on the real Quadra, and it is therefore probable that the spatial resolution of the WT-PET is also being overestimated. This overestimation is potentially due to the use of iterative reconstruction instead of filtered backprojection, even though a warm background was used.

The scatter fraction of the WT-PET is approximately 5–6% lower than that of the Quadra-like TB-PET. This variation may be attributed to the impact of utilizing the bed in the Quadra-like TB-PET. The different offsets of the line source in the WT-PET did not significantly affect the obtained value, nor did the application of the 85 MRD cut to the Quadra-like TB-PET.

Considering the IQ phantom analysis, the larger spheres produced similar CRC values in both scanners, whereas the two smallest spheres result in considerably higher CRC values for the WT-PET. It should be noted that this may in part be due to the limited projection angles, which causes an elongation of the spheres and produces a non-uniform background near the edges of the IQ phantom.

Certain regions of the WT-PET FOV may be more problematic for lesion detectability than others, not only because of reduced sensitivity, but also due to fewer projection angles being available, such as closer to the detector panels or further toward the axial end of the scanner. The most critical zones are the corners. To minimize these effects in patients, the panel design was chosen to be clearly larger than the largest patient in a sizeable database. Additionally, Fig. 12 visually shows that even lesions closer to the edge of the FOV remain reasonably detectable compared to more centered lesions.

These artifacts could be reduced by, e.g., sinogram filling methods such as the constrained Fourier space method [33] or deep learning [34]. This would, however, require binning of the data rather than working with listmode events. Another option would be image-to-image deep learning for limited angle artifact removal [35]. The large size of the angular gap remains a challenge; however, its impact is significantly reduced by the inclusion of time-of-flight information, as shown in Fig. 13.

The current design of the WT-PET does not incorporate a CT component, which is normally used both for attenuation correction and providing additional anatomical information for physicians. There are multiple options for CT-less attenuation correction, including the use of transmission sources [36], estimating attenuation coefficients from TOF emission data [37], or deep learning-based attenuation correction [38]. In order to provide anatomical information, some form of CT is, however, required. This is still an active area of study.

Table 5 describes the main design and performance characteristics of the WT-PET and Quadra-like TB-PET. As summarized there, the WT-PET shows good performance and based on the amount of required scintillator materials and SiPM covered area (as a metric to estimate the final construction cost of the scanner), it can be considered as a cost-efficient alternative option for total-body PET, although some hurdles such as limited angle artifacts and CT-less attenuation correction are still ongoing research.

**Table 5:** Geometrical and performance characteristics comparison between WT-PET (simulation) and Quadra (simulation and experimentally based results)

Scanner	WT-PET	Quadra-like TB-PET (Simulation)		Quadra PET/CT (Experimental)	
		MRD 85	MRD 322	MRD 85	MRD 322
Average FWHM @ (1,0,0) (mm)	1.37	2.68	2.67	3.51	–
Sensitivity @ Center (cps/kBq)	154.0	87.0	179.7	82.6	175.3
Scatter fraction %	30.72	34.80	36.18	36	37
Scintillation crystal volume (m⌃3)	0.0224	0.049807			
Sum of SiPM covered area (m⌃2)	1.40	2.49			

## Conclusion

The WT-PET is being developed as a novel design to achieve cost-efficient large AFOV scanners. Based on the results presented in this study, it achieves similar sensitivity to the Quadra-like TB-PET at a lower component cost (about 2–3 x), while having higher spatial resolution across the FOV due to its use of DOI-capable monolithic detectors. On the other hand, the flat panel design does introduce some limited angle artifacts in limited parts of the FOV as apparent in the IQ phantom reconstruction. Although lesion detection remains possible, as demonstrated by the introduction of additional lesions at the posterior and anterior edges of the IQ phantom, it is imperative to acknowledge that the detection of borderline-detectable lesions may be negatively impacted. Therefore, addressing and removing these artifacts should be considered a prerequisite for the clinical application of the scanner, presenting an open area for future research and optimization.

## Data Availability

The data used and/or analyzed during the current study are available from the corresponding author upon request.
